# Impact of Missing Values in the Self-Administered Washington Group Short Set: A Cross-Sectional Study Using Secondary Data of a National Survey of Persons With Disabilities in Japan

**DOI:** 10.7759/cureus.87388

**Published:** 2025-07-06

**Authors:** Takashi Saito, Kumiko Imahashi

**Affiliations:** 1 Department of Social Rehabilitation, Research Institute of National Rehabilitation Center for Persons with Disabilities, Tokorozawa, JPN

**Keywords:** disability, disability statistics, missing values, multiple imputation, washington group short set questionnaire on functioning

## Abstract

Introduction: The Washington Group Short Set on Functioning (WGSS), consisting of six questions on basic functioning, is recommended for interviewer-administered surveys. While self-administration is a practical alternative, its impact on response accuracy is poorly understood. This study aimed to quantify the extent of missing values in responses to the self-administered WGSS and enhanced WGSS (WGSSE), consisting of the six questions of the WGSS and four additional questions on basic functioning, and to examine the association between respondents’ disability-related characteristics and missing values.

Methods: In this cross-sectional study using nationally representative survey data from individuals with disabilities in Japan (n=12,822), the individual and overall percentages of missing values for the WGSS- and WGSSE-related questions were calculated. Binomial logistic regression was used to analyze the relationship between possession of disability certificates, the independent variable, and the missingness of disability status defined by the WGSS and WGSSE, the dependent variable.

Results: Non-response rates for individual WGSS- and WGSSE-related questions were 9.71-30.69%. Missingness of disability status, as defined by the WGSS and WGSSE, which was attributed to missing values for the individual questions, was observed in 16.94% and 24.32% of the participants, respectively. After adjusting for confounders, possession of a disability certificate was significantly associated with the missingness of disability status, as defined by the WGSS and WGSSE (adjusted odds ratio: 1.176-2.931).

Conclusion: Self-administration of the WGSS resulted in a significant proportion of missing responses among individuals with disabilities. Fewer individuals with disability certificates may be represented in disability statistics if missing data are excluded, potentially underestimating disability prevalence by up to approximately 20%. Stakeholders in Japan should consider these drawbacks when interpreting and using the self-administered WGSS data. Further research is needed to explore the characteristics of self-administered WGSS data.

## Introduction

The Washington Group Short Set on Functioning (WGSS), a simple questionnaire comprising six questions on functional difficulties, is widely used to measure disability internationally and generate comparable and disaggregated disability statistics. These statistics are crucial for highlighting disparities in public health and social development between people with and without disabilities [1]. Many countries and international organizations use the WGSS to monitor disability-related challenges and policies [2-4].

The WGSS developers recommend interviewer-assisted administration of WGSS [5]. This method helps maintain the representativeness of disability statistics, which can be compromised by incomplete responses, even in representative samples [6]. Interviewer administration allows for respondent guidance and encourages questionnaire completion [7], making it suitable for surveys involving people with functional impairments. Despite higher costs and time demands compared to self-administration, it remains the most common method for collecting disability data, as it minimizes the likelihood of missing data [6].

In 2022, the WGSS was included in Japan's large-scale, nationally representative survey of individuals with disabilities [8]. The survey is known as the Seikatsu no Shizurasa Chosa, which translates to the Comprehensive Survey of Persons With Difficulties (CSPD) [8]. Integrating the WGSS into the CSPD presents an opportunity to produce internationally comparable disability statistics, aiding policymakers and service providers in designing effective health and welfare services. However, caution is needed in interpreting the data because the CSPD is administered in self-administration mode. Self-administration has several advantages, including requiring less time and financial cost for conducting the survey. Conversely, as a possible drawback, a significant proportion of respondents may leave questions unanswered [9]. This may potentially bias statistics and underestimate disability prevalence by systematically excluding data from more physically or mentally vulnerable individuals, and may consequently influence disability-related policies and service delivery strategies. 

The self-administered WGSS is used particularly in Western developed countries [10,11]. Given its practicality [1], the adaptation of self-administered WGSS would expand to countries other than Western developed countries. However, its impact on response completeness and the accuracy of disability statistics is not well understood. Knowledge of the potential impact is crucial for survey design and data interpretation.

To address this knowledge gap, we conducted a cross-sectional study using secondary data from the CSPD. The aims of this study were twofold. First, we quantified missing values in the self-administered WGSS responses among individuals with disabilities in Japan. Second, we explored the relationship between missing values and respondents’ disability-related characteristics to understand who might be excluded from disability statistics due to incomplete data.

## Materials and methods

This cross-sectional observational study used secondary data from the CSPD in 2022. Data were obtained with permission from the Ministry of Health, Labor, and Welfare of Japan (MHLW). Because statistical analyses presented in this study were conducted by the authors, there may be some discrepancies compared to the official MHLW-published data. The Ethics Committee of the National Rehabilitation Center for Persons with Disabilities approved the study protocol (Approval No. 2024-069). As this study used secondary data, the requirement for informed consent was waived.

Overview of the Comprehensive Survey of Persons With Difficulties

The CSPD is a cross-sectional, nationally representative survey that focuses exclusively on individuals with disabilities. Conducted every five years since 2011, the most recent survey took place between December 1st and 22nd, 2022. The questionnaire covered various topics, including demographic information, living conditions, health and disability status, possession of disability certificates, and use of health and welfare services. The full questionnaire is available on the MHLW website (in Japanese) [12]. Study participants were community-dwelling individuals with disabilities.

Study participants were chosen from approximately 5,400 randomly selected stratified census tracts across Japan [8]. Trained survey staff visited every household within the selected tracts to enumerate eligible study participants. Eligibility criteria included individuals holding any disability certificate (physical, intellectual, or mental disorder) and those without such certificates but diagnosed with intellectual disability, developmental disorder (e.g., autism, attention deficit hyperactivity disorder, or learning disorders), higher brain dysfunction, or an incurable disease. It also included those with any functional difficulties defined by the WGSS or its enhanced version (WGSSE, described later) or prolonged conditions requiring medical devices or special education support.

During the house-to-house visits for the enumeration of study participants, a household member was asked by trained survey staff whether any household member met the eligibility criteria. This enumeration was conducted based on self-reported information from household members; no verification using medical records or registry information was made. If any eligible household members were present, a self-administered questionnaire was provided, and eligible individuals were asked to complete it by themselves and return it by mail [12]. No information on the enumeration rate and enumerated study participants’ characteristics was available.

Proxy responses, typically from family members, were permitted if enumerated study participants had difficulty completing the questionnaire. Special accommodations (e.g., questionnaires in Braille or with interpreters) could be provided by local municipalities upon request from enumerated study participants or their family members, although the extent of support provided varied. No detailed statistics are available on how many enumerated study participants requested and received such accommodations and what types of accommodations were provided for them.

A total of 24,427 individuals were enumerated as eligible study participants and provided with the questionnaire. Of these, 14,631 participants returned the questionnaires (response rate: 59.9%). After data validation, 14,079 responses were deemed valid [8]. These data were provided to the authors by the MHLW for analysis.

Variables

The variables extracted from the CSPD questionnaire included demographic data (age and sex), proxy response information, WGSS-related data, and disability characteristics.

A proxy response-related question, which appeared as the first question in the CSPD questionnaire, was presented as follows: Who answered the questions? Response options are “study participant him/herself,” “proxy who confirm answers of study participant, who was able to communicate with the proxy but not able to fill out answers by him/herself,” and “proxy who infers answers of study participant, who was not able to communicate with the proxy.”

WGSS-related data comprised 10 questions on functional difficulties [13]. Of these, six questions (difficulty seeing, hearing, walking or climbing stairs, remembering or concentrating, self-care, and communication) were common to both the WGSS and WGSSE. The remaining four questions were specific to the WGSSE and addressed two types of upper-body activities (arm/shoulder and hand/wrist) and two affective issues (anxiety and depression). Affective-related questions were further categorized into sub-questions addressing both frequency and severity. The WGSS-related specific questions, both of the original English and Japanese versions, are presented in Appendices 1, 2.

For the six common questions and the two upper-body questions, the response options were as follows: “no difficulty,” “some difficulty,” “a lot of difficulty,” or “cannot do at all.” The response options for the frequency of affective-related issues were “daily,” “weekly,” “monthly,” “a few times a year,” or “never.” For severity, the options were “a little,” “a lot,” “somewhere in between a little and a lot,” or “don’t know.” The “don’t know” response option for severity is unique to the Japanese version, as it is not available in the original English version [13].

Disability-related characteristics include information on disability certificates. In Japan, three types of certificates officially recognize a disability with varying degrees of severity: i.e., physical disability, intellectual disability, and mental disorder certificates. These certificates are required to receive social welfare services, rehabilitation, and financial support from both local and central governments in Japan. The possession of the disability certificate has been used as a classic indicator of disability status and used to estimate the prevalence of people with disabilities in Japan for decades [14]. The physical disability certificates are issued to individuals with permanent physical disabilities that affect areas such as vision, hearing, speech, limbs, organs, and the immune system. These certificates are categorized into six levels based on the severity of disability. The intellectual disability certificates are issued to individuals with intellectual disabilities. The number of categories may vary by local government; however, they are typically divided into three to four levels of severity. The mental disorder certificates are issued to individuals with mental disorders (e.g., schizophrenia, mood disorders, epilepsy, drug addiction, higher brain dysfunction, autism, or learning disorders). The severity of the disorder determines eligibility.

Definition of disability status and missingness of the disability status

The disability status was defined following the Washington Group on Disability Statistics guidelines [15]. Briefly, an individual who answered “a lot of difficulty” or “cannot do at all” to at least one WGSS- or WGSSE-related question was considered to have a disability. Conversely, an individual who answered “no difficulty” or “some difficulty” to all WGSS- or WGSSE-related questions was considered not to have a disability.

Missingness of the disability status was defined as when the disability status could not be judged due to non-responses (missing) on the individual WGSS- or WGSSE-related questions. Specifically, when all WGSS- or WGSSE-related questions were left as non-responses (missing), the case was considered as missingness of the disability status. Furthermore, if some of the WGSS- or WGSSE-related questions were left as non-responses but other questions had answers of “no difficulty” or “some difficulty,” the disability status could not able to be judged; i.e., the individual could be judged as “having disability” or “not having disability” depending on the true answer to the non-responded questions. Therefore, these cases are also considered missingness.

Inclusion and exclusion criteria for study analysis

The WGSS is recommended for individuals aged six years and older [4], while the WGSSE is recommended for those aged 18 years and older [13]. To include only individuals who are eligible for using both the WGSS and WGSSE, the inclusion criteria for the study were individuals aged 18 years and older. Individuals with missing sex or age data were excluded. Data based on proxy responses were included in the analysis.

Statistical analysis

All statistical analyses were performed using IBM SPSS Statistics for Windows, Version 28 (Released 2021; IBM Corp., Armonk, New York, United States). Statistical significance was defined as p<0.05.

First, we conducted a descriptive analysis to determine the characteristics of the eligible data. Second, to quantify the extent of missingness in the responses, we calculated the total number and percentage of missing responses for the individual WGSS- and WGSSE-related variables. Furthermore, the occurrence rate of missingness of disability status defined by the WGSS and WGSSE was calculated. Lastly, for analyzing the confounding factors adjusted relationship between respondents’ disability-related characteristics and the missingness of their disability status, binomial logistic regression analyses using the forced entry method were conducted. Specifically, the missingness of the disability status defined by the WGSS was the dependent variable, whereas possession of each disability certificate was the independent variable. Sex, age, and proxy response information were included as confounding factors. When conducting the binomial logistic regression analyses, data on disability certificates and proxy responses that had missing values were excluded; complete case analysis was conducted. A similar binomial logistic regression analysis in which the missingness of the disability status defined by the WGSSE was used as a dependent variable was conducted. As a sensitivity analysis, the same analyses were conducted using a different definition of missingness of the disability status. The missingness of the disability status was alternatively defined as when any of the WGSS-related questions were left as non-responses (missing). The case was considered as a missingness of the disability status in the sensitivity analysis.

## Results

Participant selection process

**Figure 1:**
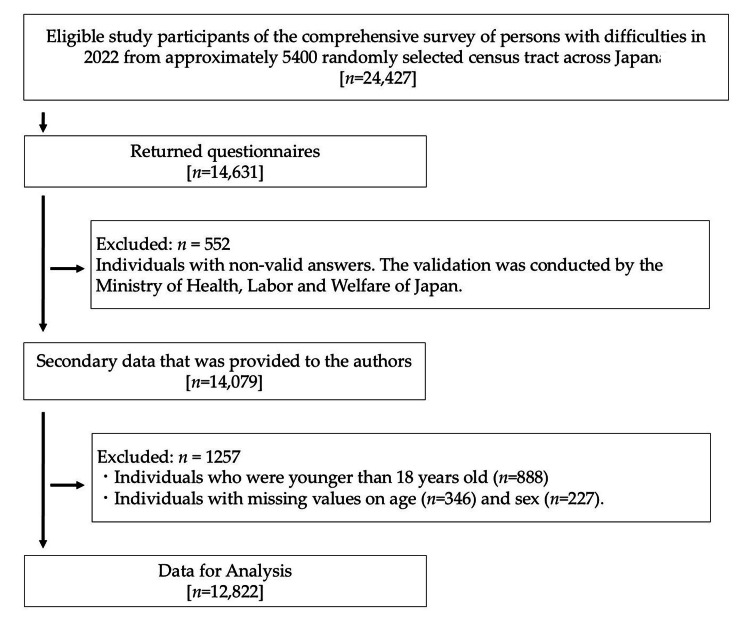
Participant selection process

The participant selection process is shown in Figure 1. Of the 14,079 valid participants, 1,257 were excluded due to meeting the exclusion criteria or missing values for age or sex, leaving 12,822 eligible participants for the analysis.

Characteristics of the study participants

Table 1 presents the characteristics of the study participants. Approximately two-thirds were aged 65 years and older, with a mean age of 67.58 ± 19.39 years. The proportion of participants identified as having a disability based on the WGSS and WGSSE criteria was 44.18% (n=5,665) and 49.34% (n=6,327), respectively. Missingness of disability status was observed in 16.94% (n=2,172) and 24.32% (n=3,118) of the participants based on the WGSS- and WGSSE-related questions, respectively. The percentage of participants with disability certificates ranged from 10.43% to 49.33%, with missing values for these variables ranging from 7.12% to 12.35%. The missing rate of disability status by age, proxy response type, and disability type was shown in Appendix 3.

**Table 1:** Characteristics of study participants by gender and age group ^†^: Proxy who confirms answers of study participant, who was able to communicate with the proxy but not able to fill out the answers by him/herself; ^††^: Proxy who inferred answers of the study participant, who was not able to communicate with the proxy; ^‡^: All six WGSS-related questions were coded as 'Missing' (non-response), alternatively, some of the WGSS-related questions were coded as 'Missing' (non-response), but others did not exceed the disability threshold, namely, two (some difficulty) or one (no difficulty); ^‡‡^: All nine WGSSE-related questions or indicators, that is, the six WGSS-related questions, the upper body indicator based on the two upper body-related questions, and two affect indicators, were coded as 'Missing' (non-response), alternatively, some of the nine WGSSE-related questions or indicators were coded as 'Missing' (non-responses), whereas others did not exceed each disability threshold. WGSS: Washington Group Short Set on Functioning; WGSSE: WGSSE enhanced

Characteristics		Men (n=6,225)		Women (n=6,597)		Total (n=12,822)	
		Number	%	Number	%	Number	%
Age (years)	18–39	819	13.16	661	10.02	1,480	11.54
	40–64	1,598	25.67	1,417	21.48	3,015	23.51
	Over 65	3,808	61.17	4,519	68.50	8,327	64.94
Self- or proxy response	Self-response	3,531	56.72	3,618	54.84	7,149	55.76
	Prox1 ^†^	1,170	18.80	1,226	18.58	2,396	18.69
	Prox2 ^††^	1,116	17.93	1,349	20.45	2,465	19.22
	Missing value	408	6.55	404	6.12	812	6.33
Physical disability certificate	Not having	2,451	39.37	3,133	47.49	5,584	43.55
	Having	3,342	53.69	2,983	45.22	6,325	49.33
	Missing value	432	6.94	481	7.29	9,13	7.12
Intellectual disability certificate	Not having	4,748	76.27	5,434	82.37	10,182	79.41
	Having	823	13.22	514	7.79	1,337	10.43
	Missing value	654	10.51	649	9.84	1,303	10.16
Mental disorder certificate	Not having	4,527	72.72	4,916	74.52	9,443	73.65
	Having	919	14.76	876	13.28	1,795	14.00
	Missing value	779	12.51	805	12.20	1,584	12.35
Disability defined by WGSS	No disability	2,556	41.06	2,429	36.82	4,985	38.88
	Disability	2,525	40.56	3,140	47.60	5,665	44.18
	Missing value^‡^	1,144	18.38	1,028	15.58	2,172	16.94
Disability defined by WGSS (enhanced)	No disability	1,777	28.55	1,600	24.25	3,377	26.34
	Disability	2,810	45.14	3,517	53.31	6,327	49.34
	Missing value^‡‡^	1,638	26.31	1,480	22.43	3,118	24.32

Percentage of participants with missing responses to the WGSS

**Figure 2:**
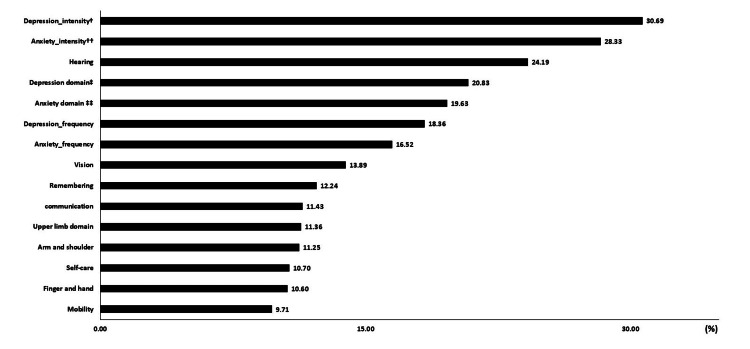
Percentages of participants with missing responses for individual WGSS- and WGSSE-related questions ^†.^ Among only those who answered the sub-questions of depression on frequency as “daily, weekly, monthly, or a few times a year” (n=10,042); ^††.^ Among only those who answered the sub-questions of anxiety on frequency as “daily, weekly, monthly, or a few times a year” (n=10,336); ^‡. ^Integrated indicator of “depression_intensity” and ” depression_frequency” following the Washington group recommendation; ^‡‡. ^Integrated indicator of “anxiety_intensity” and ” anxiety_frequency” following the Washington group recommendation WGSS: Washington Group Short Set on Functioning; WGSSE: WGSSE enhanced

Figure 2 illustrates the percentage of participants with missing responses to individual WGSS- and WGSSE-related questions. Of the five questions with the highest proportion of missing values, four pertained to affect-related questions. On average, 13.70% and 16.49% of the WGSS- and WGSSE-related questions, respectively, had missing values.

Relationships between disability certificates and missingness of disability status as defined by the WGSS

Table 2 presents the results of the binomial logistic regression. After adjusting for age, sex, and proxy response, possession of disability certificates was found to be significantly correlated with the missingness of disability status as defined by the WGSS (adjusted odds ratios (ORs) ranging from 1.198 to 2.931). Similar relationships, except for the physical disability certificate, were observed between possession of disability certificates and the missingness of disability status as defined by the WGSSE (adjusted ORs: 1.176-1.951). The results of the sensitivity analysis are shown in Appendix 4. The sensitivity analysis with other definitions of the missingness of the disability status revealed similar results.

**Table 2:** Binomial logistic regression analysis, controlled for sex, age, and proxy response, for missingness of disability status defined by the WGSS and the WGSSE † When conducting the binomial logistic regression analyses, data with missing values on sex, age, proxy response, and disability certificates were excluded; i.e., complete case analysis was conducted; †† Missingness of the disability status was defined as when the disability status could not be judged due to non-responses (missing) on the individual WGSS- or WGSSE-related questions. Specifically, when all the WGSS- or WGSSE-related questions were left as non-responses (missing), the case was considered as missingness of the disability status. Furthermore, if some of the WGSS- or WGSSE-related questions were left as non-responses but other questions had answers of “no difficulty” or “some difficulty,” the disability status could not be judged; i.e., the individual can be judged as “having disability” or “not having disability” depending on the true answer to the non-responded questions. Therefore, these cases are also considered missingness. 95% CI: 95% confidence interval; WGSS: Washington Group Short Set on Functioning; WGSSE: WGSSE enhanced

Details	Missingness of the disability status†† (Adjusted odds ratio (P-value) (95% CI))	
	Disability status (defined by WGSS)	Disability status (defined by WGSSE)
Physical disability certificate†		
Not holding	Reference	Reference
Holding	1.340	1.024
	(P<0.001)	(P=0.617)
	(1.201–1.496)	(0.932–1.126)
Intellectual disability certificate†		
Not holding	Reference	Reference
Holding	2.931	1.951
	(P<0.001)	(P<0.001)
	(2.455–3.500)	(1.664–2.287)
Mental disorder certificate†		
Not holding	Reference	Reference
Holding	1.198	1.176
	(P=0.022)	(P=0.014)
	(1.026–1.399)	(1.034–1.337)

## Discussion

To the best of our knowledge, this is the first study to estimate the impact of self-administration on WGSS-based disability statistics using a representative national sample of individuals with disabilities. Our findings showed that the self-administered versions of the WGSS and WGSSE resulted in approximately 17% and 24% missingness for disability status, respectively. This suggests that if individuals with missing data were excluded from the disability statistics, the disability prevalence would be underestimated, potentially underestimating the disability prevalence by up to approximately 20%. Moreover, possession of disability certificates was a significant contributing factor to the missingness of disability status as defined by both the WGSS and WGSSE. This implies that disability statistics excluding participants with missing disability status may be biased, as they tend to underrepresent individuals with disability certificates, i.e., those who are more vulnerable. Therefore, stakeholders in Japan must interpret or utilize disability statistics based on the WGSS and WGSSE in CSPD, with consideration of this potential underestimation and bias. Further research is needed to explore the characteristics of self-administered WGSS data.

Missing values on the self-administered WGSS

In this study, the average occurrence rates of missing values for the individual WGSS and WGSSE questions were 13.70% and 16.49%, respectively, which were notably higher than those observed in the general population. A previous study [9], which analyzed data from 41,540 participants in a representative sample of the general Japanese population, reported missing values for six self-administered WGSS questions, ranging from 1.92% to 5.51%, with an average of 2.62%. The disparity between these findings likely indicates the unique barriers or challenges faced by individuals with disabilities in completing the WGSS in a self-administered mode.

A higher percentage of missing values was particularly evident in the affect-related questions. The precise reasons for the higher missing-value rates remain unclear. One possible explanation, however, is that questions related to mental health may be more sensitive [16] than other questions on specific behaviors or activities, such as mobility, upper-body activity, or self-care. This may have led to higher non-response rates due to hesitancy in answering [17], even when administered in a self-administered mode.

Disability certification and the missingness of disability status

This study revealed a general association between possession of a disability certificate and missingness of disability status based on the WGSS and WGSSE. This suggests that individuals with vulnerabilities, such as those holding disability certificates, may disproportionately face more difficulty in completing the self-administered WGSS. Our findings align with those of Peyre et al. [18], who examined the predictors of missing responses in the self-administered SF-36, a quality-of-life indicator. Their study identified vulnerability factors, including advanced age, poor health status, and lower educational attainment, as predictors of missing data. This consistency implies that missing responses among the vulnerable populations in self-administered surveys are a generic issue. Stakeholders in Japan must interpret and utilize disability statistics based on the self-administered WGSS and WGSSE in CSPD, with consideration of this potential underestimation and bias.

Limitations and strengths

This study had several limitations and strengths. First, our findings are based on a Japanese survey with unique characteristics. Although the findings may be a reference for stakeholders in other countries, the generalizability of the findings to other countries’ contexts is not justified. Second, other factors that potentially impact the response rates were not examined in the current study, e.g., overall data collection process, structure of the questionnaire (e.g., questionnaire length and order of response options [19-21]), proxy responses, and other disability-related factors. Further research taking into account these factors is needed to gain robust knowledge on the characteristics of the self-administered WGSS and WGSSE in the Japanese population. Third, a significant number of eligible respondents did not return the questionnaire (response rate: 59.9%). Furthermore, we excluded data with missing values when conducting the binomial logistic regression. These non-respondents may have represented a more vulnerable population, introducing potential selection bias and underestimating the impact of self-administration on the WGSS- and WGSSE-based disability statistics.

Conversely, a key strength of this study is the use of secondary data from a nationally representative survey focused exclusively on individuals with disabilities in Japan. Although the response rate was not satisfactory, the study findings based on more than ten thousand data points, exclusively focusing on Japanese individuals with disabilities, can be an informative reference for stakeholders in disability-related sectors.

## Conclusions

The self-administered WGSS in the CSPD showed a significant number of missing values among individuals with disabilities. Excluding respondents with missing data from the disability statistics would result in bias, particularly underrepresenting those with disability certificates. Stakeholders in Japan should consider these drawbacks when interpreting and using the self-administered WGSS data in the CSPD. Further research is needed to explore the characteristics of self-administered WGSS data.

## Appendices

Appendix 1

**Table 3:** WGSS 1) Washington Group on Disability Statistics. Analytic Guidelines: Creating Disability Identifiers Using the Washington Group Short Set on Functioning - Enhanced (WG-SS Enhanced) SPSS Syntax. Available from: https://www.washingtongroup-disability.com/fileadmin/uploads/wg/WG_Document__7A_-_Analytic_Guidelines_for_the_WG-SS_Enhanced__SPSS_.pdf (cited on May 19, 2025). 2) Ministry of Health, Labour and Welfare of Japan. Available online: https://www.mhlw.go.jp/toukei/list/dl/seikatsu_chousa_r04.pdf (as of May 19, 2025) (in Japanese) [12]. ^† ^Answer options “Refused,” “Not ascertained,” and “Don’t know” were not available in the Japanese version. WGSS: Washington Group Short Set on Functioning; WGSSE: WGSSE enhanced

Original version (English) ^1)^		Japanese version used in the Comprehensive Survey of Persons with Difficulties ^2)^	
Vision		視覚	
Do you have difficulty seeing even if wearing glasses?	No difficulty / Yes, Some difficulty / Yes, A lot of difficulty / Cannot do at all / Refused^†^ / Not ascertained^†^ / Don’t know^†^	眼鏡をしても見えにくいといった苦労はありますか	苦労はありません / 多少苦労します / とても苦労します / 全くできません
Communication		コミュニケーション	
Using your usual language, do you have difficulty communicating (for example, understanding or being understood by others)?	No difficulty / Yes, Some difficulty / Yes, A lot of difficulty / Cannot do at all / Refused^†^ / Not ascertained^†^ / Don’t know^†^	通常の言語をつかってのコミュニケーション（たとえば、人の話を理解したり、人に話を理解させることなど）が難しいといった苦労はありますか	苦労はありません / 多少苦労します / とても苦労します / 全くできません
Hearing		聴覚	
Do you have difficulty hearing even if using a hearing aid?	No difficulty / Yes, Some difficulty / Yes, A lot of difficulty / Cannot do at all / Refused^†^ / Not ascertained^†^ / Don’t know^†^	補聴器を使用しても聞きりにくいといった苦労はありますか	苦労はありません / 多少苦労します / とても苦労します / 全くできません
Cognition		認知	
Do you have difficulty remembering or concentrating?	No difficulty / Yes, Some difficulty / Yes, A lot of difficulty / Cannot do at all / Refused^†^ / Not ascertained^†^ / Don’t know^†^	思い出したり集中したりするのが難しいといった苦労はありますか	苦労はありません / 多少苦労します / とても苦労します / 全くできません
Self-care		セルフケア	
Do you have difficulty with (self-care, such as) washing all over or dressing?	No difficulty / Yes, Some difficulty / Yes, A lot of difficulty / Cannot do at all / Refused^†^ / Not ascertained^†^ / Don’t know^†^	身体を洗ったり衣服を着るような身の回りのことが難しいといった苦労はありますか	苦労はありません / 多少苦労します / とても苦労します / 全くできません
Mobility		移動	
Do you have difficulty walking or climbing stairs?	No difficulty / Yes, Some difficulty / Yes, A lot of difficulty / Cannot do at all / Refused^†^ / Not ascertained^†^ / Don’t know^†^	歩いたり階段を上るのが難しいといった苦労はありますか	苦労はありません / 多少苦労します / とても苦労します / 全くできません

Appendix 2

**Table 4:** WGSSE 1) Washington Group on Disability Statistics. Analytic Guidelines: Creating Disability Identifiers Using the Washington Group Short Set on Functioning - Enhanced (WG-SS Enhanced) SPSS Syntax. Available from: https://www.washingtongroup-disability.com/fileadmin/uploads/wg/WG_Document__7A_-_Analytic_Guidelines_for_the_WG-SS_Enhanced__SPSS_.pdf (cited on May 19, 2025). 2) Washington Group on Disability Statistics. The Washington Group Short Set on Functioning Enhanced: Question Specifications. Available from: https://www.washingtongroup-disability.com/fileadmin/uploads/wg/Documents/Questions/WG_Implementation_Document__4C_-_WG-SS_Enhanced_Question_Specifications.pdf (cited on May 19, 2025). 3) Ministry of Health, Labour and Welfare of Japan. Available online: https://www.mhlw.go.jp/toukei/list/dl/seikatsu_chousa_r04.pdf (cited on May 19, 2025) (in Japanese) [12]. ^†^ Answer options “Refused,” “Not ascertained,” and “Don’t know” were not available in the Japanese version. If no answer was provided (the answer field was blank), the answer was considered a “non-response.” ^††^ Answer option of “Don’t know” was not available in the original English version. However, the answer was only available in the Japanese version. WGSS: Washington Group Short Set on Functioning; WGSSE: WGSSE enhanced

Original version (English) ^1,2)^		Japanese version used in the Comprehensive Survey of Persons with Difficulties ^3)^	
Vision		視覚	
Do you have difficulty seeing even if wearing glasses?	No difficulty / Yes, Some difficulty / Yes, A lot of difficulty / Cannot do at all / Refused^†^ / Not ascertained^†^ / Don’t know^†^	眼鏡をしても見えにくいといった苦労はありますか	苦労はありません / 多少苦労します / とても苦労します / 全くできません
Communication		コミュニケーション	
Using your usual language, do you have difficulty communicating (for example, understanding or being understood by others)?	No difficulty / Yes, Some difficulty / Yes, A lot of difficulty / Cannot do at all / Refused^†^ / Not ascertained^†^ / Don’t know^†^	通常の言語をつかってのコミュニケーション（たとえば、人の話を理解したり、人に話を理解させることなど）が難しいといった苦労はありますか	苦労はありません / 多少苦労します / とても苦労します / 全くできません
Hearing		聴覚	
Do you have difficulty hearing even if using a hearing aid?	No difficulty / Yes, Some difficulty / Yes, A lot of difficulty / Cannot do at all / Refused^†^ / Not ascertained^†^ / Don’t know^†^	補聴器を使用しても聞きりにくいといった苦労はありますか	苦労はありません / 多少苦労します / とても苦労します / 全くできません
Cognition		認知	
Do you have difficulty remembering or concentrating?	No difficulty / Yes, Some difficulty / Yes, A lot of difficulty / Cannot do at all / Refused^†^ / Not ascertained^†^ / Don’t know^†^	思い出したり集中したりするのが難しいといった苦労はありますか	苦労はありません / 多少苦労します / とても苦労します / 全くできません
Self-care		セルフケア	
Do you have difficulty with (self-care, such as) washing all over or dressing?	No difficulty / Yes, Some difficulty / Yes, A lot of difficulty / Cannot do at all / Refused^†^ / Not ascertained^†^ / Don’t know^†^	身体を洗ったり衣服を着るような身の回りのことが難しいといった苦労はありますか	苦労はありません / 多少苦労します / とても苦労します / 全くできません
Mobility		移動	
Do you have difficulty walking or climbing stairs?	No difficulty / Yes, Some difficulty / Yes, A lot of difficulty / Cannot do at all / Refused^†^ / Not ascertained^†^ / Don’t know^†^	歩いたり階段を上るのが難しいといった苦労はありますか	苦労はありません / 多少苦労します / とても苦労します / 全くできません
Upper body		上肢機能	
Do you have difficulty raising a 2-liter bottle of water or soda from waist to eye level?	No difficulty / Yes, Some difficulty / Yes, A lot of difficulty / Cannot do at all / Refused^†^ / Not ascertained^†^ / Don’t know^†^	2リットルの水かソーダのボトルを腰から目の高さに持ち上げることが難しいといった苦労はありますか	苦労はありません / 多少苦労します / とても苦労します / 全くできません
Do you have difficulty using your hands and fingers, such as picking up small objects, for example, a button or pencil, or opening or closing containers or bottles?	No difficulty / Yes, Some difficulty / Yes, A lot of difficulty / Cannot do at all / Refused^†^ / Not ascertained^†^ / Don’t know^†^	手と指を使って、ボタンや鉛筆のように小さいものをつまんだり容器や瓶を開け閉めするのが難しいといった苦労はありますか	苦労はありません / 多少苦労します / とても苦労します / 全くできません
Affect (Anxiety)			
How often do you feel worried, nervous, or anxious?	Daily / Weekly / Monthly / A few times a year / Never / Refused^†^ / Not ascertained^†^ / Don’t know^†^	心配、緊張、不安等をどのくらいの頻度で感じますか。	毎日 / 週に1回程度 / 月に1回程度 / 年に2,3回程度 / 全くない
Thinking about the last time you felt worried, nervous, or anxious, how would you describe the level of these feelings?	A little / A lot / Somewhere in between a little and a lot^††^	最近感じた心配、緊張、不安等の程度はどのくらいでしたか	すこし / ひどく / かなり / わからない
Affect (Depression)			
How often do you feel depressed?	Daily / Weekly / Monthly / A few times a year / Never / Refused^†^ / Not ascertained^†^ / Don’t know^†^	気分が落ち込むことがどのくらい頻繁にありますか	毎日 / 週に1回程度 / 月に1回程度 / 年に2,3回程度 / 全くない
Thinking about the last time you felt depressed, how depressed did you feel?	A little / A lot / Somewhere in between a little and a lot^††^	最近気分が落ち込んだ時の程度はどのくらいでしたか	すこし / ひどく / かなり / わからない

Appendix 3

**Table 5:** WGSS and WGSSE by age, sex, proxy response, and disability type ^†^: Proxy who confirm answers of study participant, who was able to communicate with the proxy but not able to fill-out the answers by him/herself; ^††^: Proxy who infer answers of study participant, who was not able to communicate with the proxy; ^‡^: All six WGSS-related questions were coded as 'Missing' (non-response), alternatively, some of the WGSS-related questions were coded as 'Missing' (non-response), but others did not exceed the disability threshold, namely, two (some difficulty) or one (no difficulty); ^‡‡^: All nine WGSSE-related questions or indicators, that is, the six WGSS-related questions, the upper body indicator based on the two upper body-relating questions, and two affect indicators, were coded as 'Missing' (non-response), alternatively, some of the nine WGSSE-related questions or indicators were coded as 'Missing' (non-responses), whereas others did not exceed each disability threshold. WGSS: Washington Group Short Set on Functioning; WGSSE: WGSSE enhanced

Details	WGSS						WGSSE					
	No-missing		Missing^‡^		Total		No-missing		Missing^‡‡^		Total	
	Number	(Row%)	Number	(Row%)	Number	(Row%)	Number	(Row%)	Number	(Row%)	Number	(Row%)
Sex												
Men	5081	(81.62)	1144	(18.38)	6225	(100)	4587	(73.69)	1638	(26.31)	6225	(100)
Women	5569	(84.42)	1028	(15.58)	6597	(100)	5117	(77.57)	1480	(22.43)	6597	(100)
Total	10650	(83.06)	2172	(16.94)	12822	(100)	9704	(75.68)	3118	(24.32)	12822	(100)
Age (years)												
18–39	1278	(86.35)	202	(13.65)	1480	(100)	1086	(73.38)	394	(26.62)	1480	(100)
40–64	2544	(84.38)	471	(15.62)	3015	(100)	2222	(73.7)	793	(26.3)	3015	(100)
Over 65	6828	(82)	1499	(18)	8327	(100)	6396	(76.81)	1931	(23.19)	8327	(100)
Total	10650	(83.06)	2172	(16.94)	12822	(100)	9704	(75.68)	3118	(24.32)	12822	(100)
Self- or proxy response												
Self-response	5716	(79.96)	1433	(20.04)	7149	(100)	4985	(69.73)	2164	(30.27)	7149	(100)
Prox1 ^†^	2124	(88.65)	272	(11.35)	2396	(100)	2031	(84.77)	365	(15.23)	2396	(100)
Prox2 ^††^	2202	(89.33)	263	(10.67)	2465	(100)	2121	(86.04)	344	(13.96)	2465	(100)
Missing value	608	(74.88)	204	(25.12)	812	(100)	567	(69.83)	245	(30.17)	812	(100)
Total	10650	(83.06)	2172	(16.94)	12822	(100)	9704	(75.68)	3118	(24.32)	12822	(100)
Physical disability certificate												
Not having	4854	(86.93)	730	(13.07)	5584	(100)	4311	(77.2)	1273	(22.8)	5584	(100)
Having	5170	(81.74)	1155	(18.26)	6325	(100)	4817	(76.16)	1508	(23.84)	6325	(100)
Missing value	626	(68.57)	287	(31.43)	913	(100)	576	(63.09)	337	(36.91)	913	(100)
Total	10650	(83.06)	2172	(16.94)	12822	(100)	9704	(75.68)	3118	(24.32)	12822	(100)
Intellectual disability certificate												
Not having	8777	(86.2)	1405	(13.8)	10182	(100)	7967	(78.25)	2215	(21.75)	10182	(100)
Having	1036	(77.49)	301	(22.51)	1337	(100)	944	(70.61)	393	(29.39)	1337	(100)
Missing value	837	(64.24)	466	(35.76)	1303	(100)	793	(60.86)	510	(39.14)	1303	(100)
Total	10650	(83.06)	2172	(16.94)	12822	(100)	9704	(75.68)	3118	(24.32)	12822	(100)
Mental disorder certificate												
Not having	8126	(86.05)	1317	(13.95)	9443	(100)	7432	(78.7)	2011	(21.3)	9443	(100)
Having	1490	(83.01)	305	(16.99)	1795	(100)	1293	(72.03)	502	(27.97)	1795	(100)
Missing value	1034	(65.28)	550	(34.72)	1584	(100)	979	(61.81)	605	(38.19)	1584	(100)
Total	10650	(83.06)	2172	(16.94)	12822	(100)	9704	(75.68)	3118	(24.32)	12822	(100)

Appendix 4

**Table 6:** Binomial logistic regression analysis, controlled for sex, age, and proxy response, for missingness of disability status defined by the WGSS and WGSSE (sensitivity analysis) ^†^ When conducting the binomial logistic regression analyses, data with missing value on sex, age, proxy response, and disability certificates were excluded; i e., complete case analysis was conducted; ^† †^ Missingness of the disability status was defined as when any of the WGSS-related questions were left as non-responses (missing). The case was considered as a missingness of the disability status. WGSS: Washington Group Short Set on Functioning; WGSSE: WGSSE enhanced

Details	Missingness of the disability status^††^ (Adjusted odds ratio (P-value) (95% CI))	
	Disability status defined by WGSS	Disability status defined by WGSSE
Physical disability certificate^†^		
Not holding	Reference	Reference
Holding	1.492	1.253
	(P<0.001)	(P<0.001)
	(1.362–1.634)	(1.159–1.355)
Intellectual disability certificate^†^		
Not holding	Reference	Reference
Holding	2.571	1.718
	(P<0.001)	(P<0.001)
	(2.193–3.014)	(1.492–1.978)
Mental disorder certificate^†^		
Not holding	Reference	Reference
Holding	1.263	1.139
	(P=0.001)	(P=0.023)
	(1.105–1.443)	(1.018–1.275)

## References

[1] Groce NE, Mont D (2017). Counting disability: emerging consensus on the Washington group questionnaire. Lancet Glob Health.

[2] (2025). An introduction to the Washington group on disability statistics question sets. https://www.washingtongroup-disability.com/fileadmin/uploads/wg/The_Washington_Group_Primer_-_English.pdf.

[3] Golden C (2025). Summary of annual activities related to disability statistics. https://www.washingtongroup-disability.com/fileadmin/uploads/wg/Documents/12a.pdf.

[4] (2025). The data collection tools developed by the Washington group on disability statistics and their recommended use. https://www.washingtongroup-disability.com/fileadmin/uploads/wg/Documents/WG_Implementation_Document__1_-_Data_Collection_Tools_Developed_by_the_Washington_Group.pdf.

[5] (2025). The Washington group on disability statistics: interviewer guidelines. https://www.washingtongroup-disability.com/fileadmin/uploads/wg/Documents/WG_Implementation_Document__8_-_Interviewer_Guidelines__February_2023_.pdf.

[6] (2025). Guidelines and principles for the development of disability statistics. https://unstats.un.org/unsd/demographic-social/Standards-and-Methods/files/Handbooks/disability/SeriesY_10-E.pdf.

[7] Loosveldt G (2008). Face-to-face interviews. International Handbook of Survey Methodology.

[8] (2025). Summary results of the Comprehensive Survey of Persons With Difficulties in 2022 (article in Japanese). https://www.mhlw.go.jp/toukei/list/dl/seikatsu_chousa_b_r04_01.pdf.

[9] Saito T, Imahashi K, Yamaki C (2024). The first use of the Washington Group Short Set in a national survey of Japan: characteristics of the new disability measure in comparison to an existing disability measure. Int J Environ Res Public Health.

[10] Biggs MA, Schroeder R, Casebolt MT (2023). Access to reproductive health services among people with disabilities. JAMA Netw Open.

[11] Ng K, Sainio P, Sit C (2019). Physical activity of adolescents with and without disabilities from a complete enumeration study (n = 128,803): school health promotion study 2017. Int J Environ Res Public Health.

[12] (2025). Questionnaire of the Comprehensive Survey of Persons With Difficulties in 2022 (article in Japanese). https://www.mhlw.go.jp/toukei/list/dl/seikatsu_chousa_r04.pdf.

[13] (2025). The Washington group short set on functioning - Enhanced (WG-SS enhanced). https://www.washingtongroup-disability.com/fileadmin/uploads/wg/Washington_Group_Questionnaire__3_-_WG_Short_Set_on_Functioning_-_Enhanced__October_2022_.pdf.

[14] Hayashi R (2022). Disability statistics in Japan -analysis of several indicators and historical trend (article in Japanese). IPSS Research Report.

[15] (2025). Analytic guidelines: creating disability identifiers using the Washington group short set on functioning - enhanced (WG-SS enhanced) SPSS syntax. https://www.washingtongroup-disability.com/fileadmin/uploads/wg/WG_Document__7A_-_Analytic_Guidelines_for_the_WG-SS_Enhanced__SPSS_.pdf.

[16] Rickwood DJ, Coleman-Rose CL (2023). The effect of survey administration mode on youth mental health measures: social desirability bias and sensitive questions. Heliyon.

[17] McNeeley S (2012). Sensitive issues in surveys: reducing refusals while increasing reliability and quality of responses to sensitive survey items. Handbook of Survey Methodology for the Social Science.

[18] Peyre H, Coste J, Leplège A (2010). Identifying type and determinants of missing items in quality of life questionnaires: application to the SF-36 French version of the 2003 Decennial Health Survey. Health Qual Life Outcomes.

[19] Rolstad S, Adler J, Rydén A (2011). Response burden and questionnaire length: is shorter better? A review and meta-analysis. Value Health.

[20] Lavidas K, Petropoulou A, Papadakis S (2022). Factors affecting response rates of the web survey with teachers. Computers (Basel).

[21] Robie C, Meade AW, Risavy SD, Rasheed S (2022). Effects of response option order on Likert-type psychometric properties and reactions. Educ Psychol Meas.

